# Supplementary material for: Topical 5% Imiquimod for the Treatment of Superficial and Nodular Periocular Basal Cell Carcinoma: A Systematic Review of Clinical Outcomes, Safety, and Treatment Strategies
Source: Cancers (Basel). 2025 Jun 24;17(13):2111. doi: 10.3390/cancers17132111 (PMC12248929; doi:10.3390/cancers17132111)
Supplement: Supplementary file 1 [file cancers-17-02111-s001.zip › cancers-3681040-supplementary.pdf]

Supplementary Table S1. Search strategy.

For Pubmed	For Web of science	For Scopus
("Imiquimod"[Mesh] OR "Imiquimod"[Title/Abstract] OR "Aldara"[Title/Abstract]) AND ("Eyelid Neoplasms"[Mesh] OR "eyelid basal cell carcinoma"[Title/Abstract]) AND ("Treatment Outcome"[Mesh] OR "recurrence"[Title/Abstract] OR "clinical efficacy"[Title/Abstract]) AND ("Surgery"[Title/Abstract] OR "Mohs"[Title/Abstract] OR "radiotherapy"[Title/Abstract] OR "cryotherapy"[Title/Abstract] OR "5-fluorouracil"[Title/Abstract])	TS=("Imiquimod" OR "Aldara") AND TS=("Eyelid Neoplasms" OR "eyelid basal cell carcinoma") AND TS=("Treatment Outcome" OR "recurrence" OR "clinical efficacy") AND TS=("Surgery" OR "Mohs" OR "radiotherapy" OR "cryotherapy" OR "5-fluorouracil")	(TITLE-ABS-KEY("Imiquimod") OR TITLE-ABS-KEY("Aldara")) AND (TITLE-ABS-KEY("Eyelid Neoplasms") OR TITLE-ABS-KEY("eyelid basal cell carcinoma")) AND (TITLE-ABS-KEY("Treatment Outcome") OR TITLE-ABS-KEY("recurrence") OR TITLE-ABS-KEY("clinical efficacy")) AND (TITLE-ABS-KEY("Surgery") OR TITLE-ABS-KEY("Mohs") OR TITLE-ABS-KEY("radiotherapy") OR TITLE-ABS-KEY("cryotherapy") OR TITLE-ABS-KEY("5-fluorouracil"))
